# Dietary supplementation with olive oil co-products rich in polyphenols: a novel nutraceutical approach in monogastric animal nutrition

**DOI:** 10.3389/fvets.2023.1272274

**Published:** 2023-10-13

**Authors:** Flavia Ferlisi, Jiayong Tang, Katia Cappelli, Massimo Trabalza-Marinucci

**Affiliations:** ^1^Department of Veterinary Medicine, University of Perugia, Perugia, Italy; ^2^Animal Nutrition Institute, Sichuan Agricultural University, Chengdu, China

**Keywords:** antimicrobials, antioxidants, feed supplementation, monogastric, olive oil co-products, polyphenols

## Abstract

In recent years, the increased demand for agri-food products to feed livestock species has stimulated research to identify novel solutions for the valorization of natural waste, according to the modern concept of a circular economy. Numerous studies have shown the use of plant-derived and agro-industrial co-products that are sources of bioactive molecules for preparing animal feeds. Supplementation with co-products derived from the extraction of olive oil (i.e., olive pomace, olive mill wastewater, olive cake and olive leaf) in diet has been widely considered in recent decades, because these wastes are produced in high quantity and their re-use represents an innovative economic and environmental strategy. Olive oil co-products are characterized by various bioactive molecules such as polyphenols, carbohydrates, proteins, and lipids. Among them, polyphenols are the nutraceuticals most studied, showing to promote health effects in both humans and animals. Olive oil co-products and their phenolic extracts have shown many beneficial and promising effects when added to the diets of monogastric animals, by improving performance parameters and maintaining the oxidative status of meat and derived products. This review provides an update on the use of olive co-products in monogastric animal (swine, poultry and rabbit) diets and their effects on the productive performance, meat quality characteristics and gut health status.

## 1. Introduction

The global consumption of olive oil has grown over the past three crop years due to its high nutritional value and health benefits, and should reach about 3.055 million tons in the 2022/2023 (1). It is known from the International Olive Council (IOC) that there are more than 800 million olive trees in the word, covering an area of 10 million hectares (2), and that the Mediterranean region accounts for 97% of global olive oil production (1). In Mediterranean countries, there are significant differences in the extraction methods of olive oil in different areas. The three-phase centrifugal extraction method is the most common; other techniques are the two-phase extraction and traditional discontinuous pressing (3). The three-phase decanter has become popular for its ability to decrease labor requirements and simultaneously enhance processing capacity and production of oil. However, it requires a large amount of energy and water during malaxation and generates large quantities of wastewater during the production process. The two-phase decanter separates the liquid part (the oil) from a moist solid part without requiring water, resulting in oil with high antioxidant and aromatic compound levels. However, the two-phase process typically necessitates higher energy consumption and entails a certain degree of loss of oil, because it remains in the pomace (4).

Each production process generates high amounts of co-products, mainly solid and liquid residues including olive cake, olive mill wastewater (OMWW) and olive pomace, which pollute the environment and create disposal problems (5). After two-phase centrifugation, the percentage of wet olive pomace, extra virgin olive oil (EVOO) and OMWW are 78.3, 18.7 and 3%, respectively. After three-phase centrifugation, the percentages of wet olive pomace, EVOO and OMWW are, respectively, 37.6, 17.4 and 45% (4).

In the last decades, innovative strategies including the reutilization of these wastes have become necessary, in order to reduce a negative impact on the ecosystem (6). Interestingly, these co-products can be regarded as excellent economic sources for their content in various beneficial compounds including proteins, lipids, fibers (lignin, cellulose, hemicelluloses, and pectins), polyphenols (phenolic alcohols, phenolic acids, secoiridoids and flavonoids) and other phytochemicals such as tocopherols, triterpenoids and carotenoids (7–9). The content of bioactive molecules differs according to the type of product, and for this reason it is important to consider their individual features and composition, in order to find the most useful applications in different fields (7).

Extra virgin olive oil include a number of polyphenols, among which the phenyl alcohols tyrosol and hydroxytyrosol and the secoiridoid oleuropein, which are abundant and have a considerable antioxidant and antimicrobial activity (10). After extracting olive oil, some olive oil remains in the liquid and solid phases, and these co-products retain about 98% of total phenolic compounds (11). Thus, co-products of olive oil, including OMWW and olive pomace, are considered an excellent source of polyphenols [3.0–50.0 g/kg of dry matter (DM)] (11).

Among the various bioactive molecules contained in olive oil co-products, polyphenols are the most recently studied and recovered molecules, which in several human and animal studies showed multifunctional effects that need to be further investigated (8).

Polyphenols occur naturally in plants either as free aglycones or as esters with polysaccharides or monosaccharides (12). The polyphenols encompass over 8,000 molecules that are characterized by phenolic rings with one or more hydroxyl groups, which are crucial in determining their antioxidant functions (13). Hydroxytyrosol and tyrosol are the major components found in OMWW that could be exploited as high-value compounds (13, 14). Oleuropein and hydroxytyrosol represent as much as 10% of the composition in fresh olive leaves (15), while tyrosol and hydroxytyrosol can also be found in leaf extract and olive cake (16). Furthermore, oleuropein is the main polyphenol in olive leaf extract (17), and dried olive pulp also contains antioxidant phenols such as oleuropein and hydroxytyrosol (10). Membrane technologies such as microfiltration, ultrafiltration and nanofiltration have recently been developed to extract phenolic compounds from olive oil co-products. This innovative method could be a valorization to make the co-products of olive oil a nutritional source of high economic value. Moreover, the novel recovery processes concerning olive oil co-products, in general, helps also to simplify their disposal and management (18).

Phenolic compounds show important biological effects, including anti-inflammatory, antioxidant, antibacterial, antiproliferative, antifungal and hypoglycaemic activities (19). In particular, based on their antioxidant and antimicrobial power, they have become increasingly important for livestock. Olive oil co-products could provide an advantage for the agri-food and zootechnical industries, and their inclusion in livestock feed may provide benefits without compromising their production traits.

The use of these products for animal feeds could be considered a contribution to the circular economy of Mediterranean countries such as Italy, Spain and Greece, which are the main olive oil producers. Moreover, agro-industrial co-products are usually less expensive compared with the feeds traditionally used (20). Regarding animal nutrition, especially for monogastric animals, there has been extensive research into the use of plant extracts high in polyphenols (21). Plant extracts can be regarded as promising alternatives to synthetic antioxidants, given the characteristics of many natural compounds they contain, especially polyphenols (21). The potent antimicrobial properties of phenolic compounds can also potentially reduce the risk of bacterial infections (22). In this regard, it should be useful be remembered that the European Union (EU) has prohibited the use of antibiotics as growth promoters in livestock since 2006 (EC Regulation No. 1831/2003), owing to the potential detrimental impacts on food safety and animal health (23, 24). The use of polyphenols as potential natural feed supplements with a role in the immunity, antimicrobial, antioxidant power and overall production performance of swine and poultry has been confirmed by *in vivo* and *in vitro* studies (21, 25). Furthermore, dietary supplementation with polyphenols could enhance food oxidative stability originating from livestock animals (26). Phenolic compounds can also be applied as natural compounds protecting animals from the bad consequences of feed component oxidation, particularly when there is an high unsaturated fatty acid content (25). This review summarizes the studies available that have examined in monogastric animals the effects of olive oil co-products and extracts as sources of beneficial bioactive molecules, including polyphenols.

## 2. Chemical composition of olive oil co-products

### 2.1. Olive pomace and olive cake

Olive pomace is the primary solid product that remains after extracting olive oil from the fruit; it is composed of oil, skin, pulp and pits and has a relatively high water content. It is a good source of beneficial molecules such as carbohydrates, proteins, lipids and polyphenols (7, 27). Olive pomace is rich in fibrous compounds such as cellulose, lignin, pectins and hemicelluloses (xyloglucans, xylans, mannans and glucoroxylans) and also contains small amounts of organic nitrogen as well as minerals (especially potassium) (7, 27).

Interestingly, this co-product is composed by oleuropeosides, polyphenols and flavonoids. The extract of olive pomace contains at least 10% of triterpenes and 2% of polyphenols (28).

Olive cake is a semi-liquid paste produced by pressing and compressing the olive pomace to extract any remaining oil. The olive cake consists of stone (18–32%), pulp, olive skin, kernel, a portion of oil, and approximately 40–60% of water. The olive cake can be regarded as a source of various phytochemicals such as peptides, flavonoids (quercetin), tocopherols and polyphenols (7). Interestingly, vitamin E is a group of exogenous antioxidants including tocopherols that is lipophilic and can work in a synergically with phenolic acids, potentiating its action (29).

Olive cakes devoid of stones exhibit a significant presence of fiber and lignin (160–557 g/kg DM), while the crude protein (CP) content is relatively low and varies from 44 to 115 g/kg DM (30, 31). Some olive cake can be used in the diets of finishing pigs after partial defatting. On dry matter basis, olive cake contains 52–92 g/kg of CP, 7.9 g/kg of Ca, 9 g/kg of total phosphorus, 122 g/kg of ether extract, 8.6 g/kg of total polyphenols (gallic acid equivalents) and 170 g/kg of lignin (32, 33). The semisolid destoned olive cake, called pâté, has an olive oil concentration varying from approximately 8 to 12%, depending on the moisture content. Pâté also has high levels of sugars, structural carbohydrates and a moderate CP content. Fatty acids in pâté mainly comprise oleic acid and polyunsaturated fatty acids (34). The main components of pâté are the following: total polyphenols 120 mg/kg, hydroxytyrosol 54 mg/kg, tyrosol 60 mg/kg, pinoresinol 3 mg/kg, and verbascoside 3.5 mg/kg (34).

### 2.2. Olive pulp

Olive pulp refers to the fleshy part of the olive fruit that is left over after the oil is extracted. It consists of fragments of olive stones, skin and a small portion of olive oil; it is what remains of the olive cake after drying. Olive pulp is rich in essential fatty acids (73% oleic acid, 13% palmitic acid and 7% linoleic acid) and has a high residual oil (35). Because of this high residual content, dried olive pulp could be a low-cost source of energy. Olive pulp contains fibers, whose contents may change depending on the processing (36). It is also composed by protein, fat, calcium, copper and cobalt, even if it does not have a high nutritional value because of its decreased digestible protein and mineral content and a high lignin concentration (36). Dried olive pulp is considered a relevant reservoir of natural antioxidants, with a high concentration of hydroxytyrosol (228.6 mg/kg DM) and oleuropein (1,007 mg/kg DM), and has antibacterial and antifungal effects (10). The proximate chemical composition of dried olive pulp includes 88–95% of DM, 7–12% of CP, 28–35% of crude fiber, 7.9–19.0 g/kg DM of total phenolic compounds, and 229 mg/kg DM of 3,4-dihydroxyphenylethanol (DHPEA) (10, 37, 38).

### 2.3. Olive mill wastewater

Olive mill wastewater is the liquid waste generated during the production of olive oil. It consists of the vegetation water of olives diluted during the extraction of oil and has a high polyphenolic content (up to 53% of olive fruit phenolic compounds) for the elevated water solubility of these molecules (39). This liquid product presents a high content of organic compounds such as proteins, lipids, carbohydrates, tocopherols, carotenoids and polyphenols (7).

The amount of OMWW produced every year in the Mediterranean region is approximately 30 million m^3^ (40). Polyphenolic powder can be extracted from OMWW by separating the solids; acidifying the solution; extracting with a solvent; purifying through filtration; and precipitating, drying and storing the isolated polyphenols (40). Different extraction processes have a greater impact on the content of phenolic compounds. The content of total polyphenols is approximately 100 mg/g, with hydroxytyrosol and tyrosol being 0.5 and 0.55 mg/g, respectively (41). In terms of g/kg DM, the content of total polyphenolic is approximately 96.6 g/kg, with hydroxytyrosol and tyrosol representing, respectively, 20.8 and 3.9 g/kg of the composition (13).

### 2.4. Olive leaf extract

Another interesting co-product of olive fruit is the olive leaf extract, which is a bitter-tasting, dark brown liquid derived from olive leaves; it represents approximately 10% of the material deriving from the olive oil press (42, 43). Olive leaves are remnants from the agricultural process of harvesting olives, where the olive trees are beaten to collect the fruits. Pruning yields about 25 kg of olive leaves per tree. The chemical composition of olive leaves consists of a high quantity of extractives (36.52%), lignin (39.6%), cellulose (5.7%) and hemicelluloses (3.8%), as well as crude proteins (ranging from 8.10 to 39.6%) (7).

Fresh olive leaves contain approximately 10% polyphenols (15), specifically oleuropein and hydroxytyrosol. After drying for 2 days at 37°C, olive leaves have up to 25 g/kg total polyphenols and 22 g/kg oleuropein (43). Deriving from the leaves of the olive tree, olive leaf extract is a dark brown liquid with a bitter taste and is also rich in polyphenols, which have a strong antimicrobial effect (44). Among them, oleuropein is the most abundant, with a concentration ranging from 60 to 90 mg/g in dried olive leaves (45).

## 3. Antimicrobial properties and effects on gut health

In plants and plant extracts, polyphenols are the main secondary metabolites widely known to have antimicrobial action (46). The antimicrobial effects of co-products from olive oil production (especially olive mill waste water and olive leaf extracts) have been related to the presence of phenolic compounds (47), among which oleuropein and hydroxytyrosol are the two most powerful bioactive molecules contained (48, 49). Many polyphenols are able to inhibit the microbial growth and also interfere with oxidative reactions (46). Oleuropein can stimulate the increase of nitric oxide inside the macrophages, protecting them from the endotoxins produced by the gram-negative bacterial species (50). Additionally, extracts can be considered more effective than insolated compounds, because each constituent can be affected by the others contained. In this way, the use of extracts, including the ones from olive oil production, is considered essential for enhancing the synergistic actions of their bioactive compounds (49), with possible applications in animal feed as natural antimicrobial compounds (51). Moreover, a greater antimicrobial activity of olive co-products such as olive leaf extract could be attributed to many phenolic metabolites, including the products of oleuropein hydrolysis (elenolic acid and oleuropein aglycon) rather than the oleuropein glucoside form (49).

The antimicrobial effect of natural extracts can be also related to their the ability to change the intestinal bacterial population (52) and/or modulating the immune response in the gut. In the body, the primary defense system against adverse microorganisms and harmful internal and external substances is the intestinal tract. However, oxidative stress can compromise this function, resulting in intestinal cell damage, apoptosis and reduction in tight junction proteins’ expression, that contribute to the function of mucosal barrier (53). Gut health is crucial for animal growth and is closely related to intestinal immune function. In farm animals, oxidative stress is often associated with gut dysbiosis and pathogenic infections, which can lead to reductions in the antioxidant capacity and increased lipid peroxidation, potentially resulting in severe conditions like sepsis (54). In broiler chickens and weaned piglets, oxidative distress may also contribute to microbial infections, causing inflammation and a reduction of feed efficiency related to a gut function impairment (13).

Interestingly, polyphenols from olive oil have been found to maintain the integrity of gut barrier by increasing the expression of genes involved in tight junction stability and modulating the oxidative state, immune and inflammatory response in the intestinal epithelium (55). Polyphenols also act during weaning stress in piglets and calves by improving not only nutrient absorption and digestion, but also intestinal barrier and intestinal microbiota function (56). Furthermore, olive oil co-products rich in phenolic compounds can induce a modification of gut microbiota populations not only by decreasing intestinal pathogens (i.e., *Helicobacter pylori* and *Escherichia coli*) (42), but also exerting a prebiotic action in the gut through an induction of the growth of helpful bacteria such as *Clostridium* (44, 57) and *Lactobacillus* (44, 58). In every animal species, particularly during the first days of life, is important to have a good microbial composition in the gut, in order to maintain homeostasis and avoid intestinal tract disorders that may occur, especially in the early period (59). In piglets, gut microbiota colonization at birth also derives from the microbiota of the maternal intestinal tract (59). For this reason, the increase of *Lactobacillus* spp. and *Bifidobacterium* spp. in the sow’s intestinal tract after dietary administration with olive oil co-products (such as olive pulp) is an important strategy to transfer these effects to the offspring (60). In laying hens, supplementation with olive oil co-products (e.g., with fermented defatted olive pomace) in early life induced an increase of the abundance of Firmicutes, Proteobacteria and Actinobacteria, also stimulating bacterial diversity. These actions can strengthen the innate immune response, help to compete with microbial pathogens and could potentially reduce the antibiotic use (61).

### 3.1. Olive cake

Dietary supplementation of piglets with an extract of olive cake partially restores intestinal villi height and reduces the depth of crypts by alleviating the lipopolysaccharide (LPS)-induced inflammatory response (53). Furthermore, in the same study the olive cake extract group had a reduced impairment of small intestine morphology, showing that this supplementation can contribute to the morphological and structural integrity of intestine by alleviating LPS-induced damage. Additionally, olive cake extract has been shown to increase the amount of the pro-biotic bacteria *Clostridium* and *Lactobacillus* by lowering the pH, displaying a beneficial effect on pig intestine (53) (Table 1).

**Table 1:** Effects of olive oil co-products on microbial populations.

Co-product	Content of polyphenols (g/kg)	Dosage (g/kg)	Species	Effect	References
Olive leaf extract	97	0.5, 1.0	Ross 308 male broilers	Total aerobic bacteria →, Total coliform bacteria ↓, *Lactobacilli* bacteria ↑	(44)
OMWPE, DOC	0.0125, 0.0625	160, 330	Ross 308 female broilers	*Campylobacter* spp. ↓	(5)
Olive cake	8.6	120	Finishing pigs	Total aerobic bacteria →, *Enterobacteria* →, *Bifidobacteria* →, *Lactobacilli* →	(32)
Olive oil cake extract	-	1.0	Weaned piglets (DLL)	*Clostriudium _sensu_scricto*_ ↑, *Lactobacillus* ↑	(53)

DLL, Duroc × Landrace × Large White; DOC, dried olive cake; GA, gallic acid; OMWPE, Olive mill wastewater polyphenolic extract. ↑, increased; ↓, decreased; →, no difference.

### 3.2. Olive mill wastewater

Bonos et al. (57) recently demonstrated that silage supplemented with OMWW significantly reduced the amounts of harmful bacteria such as *Clostridium* spp., *E. coli*, and *Campylobacter jejuni*, suggesting a good correlation between gastrointestinal tract modifications and bird health. Olive mill vegetation water is also able to improve meat microbial quality (62). In poultry, dietary treatment with a phenolic extract from OMWW decreased *Campylobacter* spp. prevalence, by controlling the spread of the bacterium when the production lifespan ended, which could reduce the risk of *Campylobacter* infection in processed poultry meat (5) (Table 1).

### 3.3. Olive leaves

In one study, olive leaf extract dietary supplementation (100 and 200 mg/kg) was more efficient in decreasing the number of total coliform bacteria and *E. coli* compared to a control diet (44). Additionally, feeding broiler chickens with an olive leaf extract reduced the level of total coliform bacteria. This extract exerted also a beneficial effect on the microflora content of broiler chicken ileum (by increasing the concentration of *Lactobacillus*) and could be used as natural antioxidant alternatives to probiotics (44). Olive leaf extracts have also been shown to successfully inhibit *in vitro* the adhesion of *C. jejuni* and hamper its colonization (63). Olive leaf extract polyphenols exhibit antimicrobial activity against various pathogenic microorganisms, particularly *E. coli*, and the combined phenolics exert antioxidant and antimicrobial effects comparable to or better than individual phenolics (64). Additionally, in broiler chickens, dietary inclusion of different levels of an olive leaf powder improved the intestinal morphology by enhancing the length and surface of villi and the depth of crypts (65).

## 4. Effects on animal performance

A current issue in livestock industry is the promotion of animal performances without increasing production costs, minimizing environmental impacts and maximizing welfare (66). In the animal body, different stress conditions including farm practices, changes of environment, transportation, lairage and fasting preceding slaughter can disrupt the balance between the antioxidant defense and the production of free radicals (67). Reactive oxygen species (ROS) and nitrogen species (RNS), the most common groups of free radicals in biological systems, are usually produced at low or moderate levels in livestock animals being exposed to the stress conditions of the extensive or rangeland production (68). The excess of ROS in the body can be detrimental. Moreover, animals with faster growth rates, and thus high metabolic rates, are more prone to oxidative stress (68), causing negative effects on livestock. Accumulating evidence suggests that oxidative stress can reduce the health status, leading to an impaired immune system and lower productivity and welfare (67).

Stress regulates the functions of the cells by inducing physiological or pathological modifications, and consequently affecting metabolism and the fate of nutrients in the body. Furthermore, oxidative stress could induce DNA, lipid and protein damage, changes that are correlated to various harmful consequences which alter animal production and performances (69). This may contribute to deterioration of the qualitative characteristics of meat products (68). Additionally, in piglets, weaning is a very stressful condition characterized by high production of free radicals, leading to all the oxidative-stress induced negative effects (70). Post-weaning stress is often linked to a decreased growth performance, as well as to an higher susceptibility to microbial infections (71, 72).

Animal performance and quality of the derived products are markedly affected in monogastrics, especially chickens (16, 28, 33, 44, 73) and pigs (43, 74, 75). On the farm, the use of antioxidants is required during animal life conditions such as reproduction and growth, in order to maintain the oxidative balance in the biological systems of both cells and tissues (68). Recent research has focused on the addition of natural antioxidants in livestock feed, in order to improve the productivity and health of animals, protecting them from the harmful effects of oxidative stress (76). More specifically, the use of phenolic compounds can reduce the oxidative stress-induced damages in animals due to the antibiotic-like action of these molecules (77). In this way, these molecules can also improve the antioxidant potential of products of animal origin, e.g., meat.

Many *in vitro* and *in vivo* animal studies have demonstrated that polyphenols present in olive oil, olive fruit and extracts (such as OMWW) exert strong biological activities, mostly-but not only exclusively-linked to their antioxidant power (78). For example, pretreatment with hydroxytyrosol protects renal cells from membrane oxidative damage induced by free radicals by improving also the morphology and biochemical functions in the proximal tubular epithelium (79). The protective action of hydroxytyrosol from EVOO has been also demonstrated against the damage induced by ochratoxins in a Madin–Darby canine kidney cell line (MDCK) and rabbit kidney cell line (RK 13) (80).

Regarding the *in vivo* effects, the use of natural plants and their extract rich in polyphenols in diet has been recently considered as a novel strategy in farm animals, in order to improve their growth and reduce mortality (81). Polyphenols can be considered as good promoters of farm animal growth by stimulating saliva, enzyme digestion, secretion of mucus and bile, and by protecting gut morphology thanks to their anti-inflammatory and anti-oxidant action (81). Moreover, the improvement of chicken growth rate is probably related to a reduction of the passage rate induced by polyphenols during digestion, which increases the digestibility of feed (38). Among the co-products of olive oil extraction, olive leaf extract supplementation in animal feed regulates the digestion and enhances digestive juices stimulating appetite and the consumption of food by the animal. This extract may additionally prevent diseases for its antibacterial and antifungal activity, and improves animal performance (73).

### 4.1. Olive pomace and olive cake

Herrero-Encinas et al. (28) demonstrated that a bioactive olive pomace extract added to chicken diets (750 mg/kg) improved animal growth given its anti-inflammatory activities and did not induce a negative effect on body weight (BW) and feed conversion ratio (FCR) (Table 2). Moreover, olive cake supplementation at 10% to broiler chicken diets significantly improved the FCR (33), while its inclusion up to 15% in laying hen diets increased feed intake (FI) and improved feed efficiency (82). Branciari et al. (5) confirmed these positive effects in poultry: they found that dietary supplementation with a dehydrated olive cake could increase live weights compared to the controls. Dietary olive cake (10%) with citric acid improved final BW and daily and total body weight gain (BWG) in broiler chickens (88). In rabbit diets, supplementation with olive cake at 30% and bentonite led to improvements of final BW, daily weight gain and FCR parameters (89). Olive cake pulp (up to 25%) added in growing rabbits did not induce harmful effects on their growth performance parameters (90). Furthermore, dietary supplementation with 82.5 and 165 g/kg of pâté olive cake led to greater BW in chickens compared with the control diet (34). A diet based on wet crude olive cake in Iberian pigs resulted in a better growth performance compared with dry olive pulp (91), and the addition of olive cake in finishing pig diets also increased FI and BWG (37). Similarly, Joven et al. (75) reported in pigs an higher consumption of feeds and higher growth rates after a dietary supplementation with 5% or 10% of olive cake (replacing an equivalent proportion of barley in the diet) (Table 2). In contrast, during the pig finishing period, dietary supplementation with 100 mg/kg of partially defatted olive cake had no effect on growth performances, showed only a tendency to increase FCR (*p* = 0.059) and significantly reduced loin depth (32).

**Table 2:** Effects of olive co-products supplementation on animal growth performances.

Co-product	Content of polyphenols (g/kg)	Dosage (g/kg)	Species	Effect	References
Olive cake	-	100, 150	Ross 308 broilers	BWG →, FI →, FCR ↓	(82)
Olive pomace extract	20	0.75	Ross 308 broilers	ADG↑, FCR ↓, ADFI→, AID →	(28)
Olive pulp	2.41	30, 60	Ross broilers	BW →, FC →, FCR →, hock burn →, feather cleanliness ↑, foot pad dermatitis ↓	(83)
Olive leaf extract	97	0.5,1.0	Ross 308 broilers	BWG ↑, FCR ↓, FI →	(44)
Olive leaf extract	4.4	5, 10, 15	Arbor acre broilers	ADG ↑, ADFI ↑, FCR ↓	(16)
Olive cake	-	50, 100	Ross broilers	BW →, FI →, FCR →, survival rate ↑	(33)
Pâté olive cake	0.17	82.5, 165	Ross 308 broilers	LW ↑, ADG ↑, FCR ↓	(34)
Olive leaf and grape	7	2	Ross 308 broilers	ADG →, ADFI →, FCR →, EBI →	(84)
Olive pulp	-	25–50, 50–50, 50–80	Cobb 500 broilers	ADG →, ADFI →, FCR ↑	(10)
Olive pulp	7.9	50, 100	Hubbard broilers	BW →, ADG →, ADFI →, FCR →, mortality →	(85)
OMWW extract	100	0.2, 0.5	Hubbard-Sasso broilers	BW →, water consumption →	(41)
Olive cake	-	50, 100, 150	Finishing pig	BW ↑, FI ↑, ADG →, FCR →, ADE ↑	(75)
Olive leaf	25	50, 100	Growing-finishing pigs	FW↓, ADG↓, ADFI↓, F/G↑, ATTD↓	(43)
Olive cake	8.6	120	Finishing pigs	BW →, ADG →, ADFI →, FCR →, LD ↓	(32)
Olive cake	-	100	Finishing Bísaro pigs	BW →, pH →, color →, CW →	(86)
Olive cake	-	50, 100	Pietrain pigs	BW ↑, FCR ↑	(87)

ADFI, average daily feed intake; ADG, average daily gain; ADE, apparent digestible energy; AID, apparent ileal digestibility; ATTD, apparent total tract digestibility; BW, body weight; BWG, body weight gain; EBI, European broiler index; FCR, feed conversion ratio; FI, feed intake; FW, feed weight; GA, gallic acid; LD, loin depth; LW, live weight; OMWW, olive mill wastewater. ↑, increased; ↓, decreased; →, no difference.

### 4.2. Olive pulp

Dietary supplementation with dried olive pulp can be considered a good strategy for feeding slow-growing broilers, as it has no adverse effects on animal productive performance, carcass weight, yield of breast, and fatty acid composition of breast meat (10). An increase in FI has been observed when supplementing broiler chicken diets with olive pulp (37). In addition, researchers reported an increase in BW and BWG of broilers subjected to heat stress after dietary treatment with olive pulp (92).

### 4.3. Olive mill wastewater

A natural polyphenolic product from OMWW included in post-weaning pig feed induced higher BW and average daily gain (ADG) (70). Furthermore, this phenolic administration improved clinical performance by decreasing the frequency of post-weaning diarrhea (70). Branciari et al. (5) demonstrated that in poultry, supplementation with an OMWW polyphenolic extract could increase final BW and carcass weight compared to the control. Sabino et al. (93) did not find differences in feed conversion efficiency after dietary supplementation with OMWW in chicken diets compared to controls, but the observed morphological changes in the jejunum of the OMWW-supplemented group suggest that this co-product could have a beneficial effect on the intestinal ecosystem.

### 4.4. Olive leaves

Researchers have demonstrated that olive leaves can be added to feeds without adversely affecting growth performance (94). The healthful effects of the olive leaf extract have been attributed to the known antioxidant, anti-microbial and anti-inflammatory activity (12) (Table 2). Paiva-Martins et al. (43) investigated the effect of the dietary addition of olive leaf extract on feed digestion, growth performance and meat qualitative characteristics of pigs (Table 2). Olive leaf supplementation improved growth performance, with a better feed-to-gain ratio by comparing with a conventional diet. Sarıca and Ürkmez (44) reported in broiler chickens an increase in BWG and a decrease in the FCR during the 6 weeks after supplementation with a composition of either 100 or 200 mg/kg of olive leaf extract (Table 2). In broiler chickens reared in humid and warm temperature, supplementation of drinking water with olive leaf extract at 15 mL/L (containing 66 mg/L oleuropein) improved BW, BWG, FI and the FCR (16). Furthermore, supplementation of growing rabbits with until 1.5 mL/kg of an olive leaf extract improved several performance parameters (final BW and the FCR) (95). The supplementation of an aqueous extract of olive leaf also increased ADG in growing rabbits (96).

## 5. Effects on meat quality

Antioxidants can contribute to animal welfare and productivity by delaying or preventing lipid oxidation through the reduction of free radical activity in meat (43). An excess of reactive oxygen species (such as ROS and RNS) reduce the quality of meat, causing many defects in flavor and taste that compromise the biological and reduce meat shelf life (67). Polyunsaturated fatty acids are highly susceptible to oxidation, and oxidation products can destroy the nutritional, chemical and sensory characteristics of meat, especially tenderness, juiciness, flavor, drip loss and shelf life (15, 43, 97). Nutritional supplementation is a novel strategy that can improve meat stability by changing the profile of fatty acids or the content of tocopherols in the muscle. Notably, the addition of anti-oxidants in animal feed is considered a useful method to increase meat stability (98). Recently, the use of natural antioxidants including polyphenols has been recommended, in order to restrict lipoperoxidation and maintain qualitative characteristics (flavor, color, tenderness) and shelf life of animal-derived products, ensuring their healthfulness for the consumers (99). Co-products deriving from olive oil could be employed as potential animal nutrients to produce high-quality meat based on their strong radical scavenger activity from its very high levels of polyphenols, including oleuropein, hydroxytyrosol and verbascoside (100). Hydroxytyrosol is able to scavenge the peroxyl radicals near the surface of the membranes, and oleuropein can interfere with their chain propagation (101). Increased dietary absorption of polyphenols can also exert a protective effect on the low-molecular-weight antioxidant tocopherol by acting as a barrier against the oxidation of lipids (68).

Oxidative stress can be evaluated with the thiobarbituric acid-reactive substance (TBARS) content, which measures lipoperoxidation due to free radical generation. Branciari et al. (34) reported the TBARS content of meat from animals fed with olive polyphenols. They found that this supplementation improved the oxidative status of the meat. Recent *in vivo* studies have reported that co-products from olive oil rich in polyphenols improve the antioxidant state and welfare of monogastric species including chickens, pigs and rabbits, as well as the quality of their meat (34). Tufarelli et al. (38) also demonstrated an improvement of antioxidant status with lower TBARS in the liver of chickens fed with a supplementation of EVOO. Additionally, a reduction in saturated fatty acids and a monosaturated fatty acids increase improves the meat chemical composition, and these effects can be induced by polyphenols from olive oil co-products (32, 75) (Table 3). The analysis of fat content in animal-based products usually consists in the evaluation of nutritional parameters including the ratio between polyunsaturated/saturated fatty acids and of n-6/n-3 fatty acids (104), which are important to ensure healthy human nutrition.

**Table 3:** Effects of olive co-products supplementation on meat quality.

Co-product	Content of polyphenols (g/kg)	Dosage (g/kg)	Species	Effect	References
OMWW extract, DOC	0.125, 0.0625	160, 330	Ross broilers	LW ↑, CW ↑, dressing percentage →	(5)
Olive leaf and grape	7	2	Ross 308 broilers	CY →, color →, drip loss →	(84)
Olive pulp	-	25–50, 50–50, 50–80	Cobb 500 broilers	SFA ↓, MUFA ↑, MDA →, cooking loss →, shear force →, pH_24_ ↓, a* →, b* → L*↑	(10)
OMWW extract	100	0.2, 0.5	Hubbard-Sasso broilers	CAT ↑, GSH ↑, TAC ↑, protein oxidation ↓, lipid peroxidation ↓	(41)
Olive pulp	7.9	50, 100	Hubbard broilers	Carcass traits →, pH_24_ ↑, FA →, L* ↑, a* ↑, b* ↓, TBARS ↓, SFA ↓, MUFA ↑, PUFA/SFA →, Lipid hydroperoxides ↓	(85)
Pâté olive cake	0.17	82.5, 165	Ross 308 broilers	pH →, drip loss →, cooking loss →, shear force →, TBARS ↓, DPPH ↑	(34)
Olive pulp	-	50	Finishing pigs	CW →, pH →, cooking loss →, b* ↓, shear force →	(74)
Olive leaf	25	50, 100	growing-finishing pigs	α-tocopherol ↑, pH →, color →, cooking loss →, drip loss ↓	(43)
Olive cake	-	50, 100, 150	Finishing pigs	CW ↑, fat depth ↓, SFA ↓, MUFA ↑, PUFA →	(75)
Olive cake	-	200, 400	pigs	MUFA ↑, OA ↑, PUFA ↓, LA ↓	(102)
Olive leaf	67	25, 50	Finishing pigs	Improvement of tocopherol content	(103)
Olive pulp	-	50	Finishing pigs	CW →, pH →, cooking loss →, b* ↓, shear force →	(74)

a*, redness; ADE, apparent digestible energy; b*, yellowness; CAT, catalase; CW, carcass weight; CY, carcass yield; DOC, dried olive cake; DPPH, 2,2-diphenyl-1-picrylhydrazyl; FA, fatty acid; GSH, glutathione; L*, lightness; LA, linoleic acid; LW, live weight; MDA, malondialdehyde; MUFA, monounsaturated fatty acids; OA, oleic acid; OMWW, olive mill wastewater; PUFA, polyunsaturated fatty acids; SFA, saturated fatty acids; TAC, total antioxidant capacity; TBARS, thiobarbituric acid reactive substances. ↑, increased; ↓, decreased; →, no difference.

### 5.1. Olive pomace and olive cake

De Oliveira et al. (105) demonstrated that in chickens olive pomace can induce a modification of the lipid composition in meat by increasing the monounsaturated fatty acids content and decreasing the amount of the saturated ones. The same co-product type (from 5 to 16%) added in rabbit diets significantly increased meat monounsaturated fatty acids, with a correlated reduction in the polyunsaturated ones (100). Moreover, a reduced meat peroxidation has been observed in the olive pomace–supplemented group, in comparison to the control (100).

Dietary supplementation of a chicken diet with a high concentration of pâté led to a reduction of TBARS values in meat (34). Increasing levels of olive cake (up to 15%) led to healthy fatty acid profiles in pig fat by promoting a linear decrease in the proportion of total saturated fatty acids and an increase in the percentage of the total monounsaturated ones (75). The addition of partially defatted olive cake in pig diets did not shown a significant effect on carcass quality, microbial counts and subcutaneous fatty acids profile, but induced a lower pH and polyunsaturated fatty acid content and higher monounsaturated fatty acid concentration in the meat (32). Additionally, the inclusion of olive cake silage in the diet (up to 40%) of Apulo-Calabrese pigs promoted a higher proportion of monounsaturated fatty acid, especially oleic acid, and a lower concentration of polyunsaturated acids (102).

### 5.2. Olive pulp

The results reported by Papadomichelakis et al. (10) confirmed that feeding broiler chickens with dried olive pulp can increase the content of monounsaturated fatty acids in meat. The addition of this olive co-product in broiler diets also induce a significant improvement in meat color, as indicated by higher meat lightness (L*) and redness (a*) values (85). Moreover, in finishing pigs, meat yellowness decreased and meat oxidation stability tended to be improved after dried olive pulp supplementation (74).

### 5.3. Olive mill wastewater

Olive mill wastewater supplementation in chicken feed can induce a decrease in the oxidation of lipids and proteins in meat without influencing its color stability. Moreover, piglets fed with an OMWW polyphenolic co-product showed lesser damages in proteins and lipids related to oxidative stress, indicated by a decrease in TBARS and protein carbonyl contents (106). The addition of a polyphenolic powder from OMWW to chicken diets reduced oxidative stress–induced damage (41). Gerasopoulos et al. (14) indicated that in piglets, feed containing polyphenolic additives from processed OMWW improved the lipid ratio and the quality of the meat. OMWW supplementation into rabbit diets also decreased *Pseudomonas* spp. growth in the meat (107).

### 5.4. Olive leaves

Olive leaves contain a large amount of polyphenols, which are strong natural antioxidants potentially able to decrease the excess of free radicals and harmful DNA modifications (41). Olive leaf extract could be used to produce high-quality meat due its very high level of polyphenols, including oleuropein (108). Supplementation of pig diets with 5% or 10% olive leaf extract significantly increased α-tocopherol content in *Longissimus dorsi* muscle and backfat compared with a control diet (43) (Table 3). Even if added at low dosages, olive leaf extract is a beneficial source of biologically-active molecules and could increase tocopherols in meat (43). In broiler chickens, dietary supplementation with olive leaf and a grape co-product (2 g/kg) changed breast color, by increasing its yellowness (b*) values and color intensity (84), compared with protected sodium butyrate. The same dietary supplementation seemed to decrease drip loss, a change related to enhanced breast meat quality (84) (Table 3). The combination of oleuropein, magnesium, betaine and vitamin E in pig diet could improve the oxidative state and maintain the stability of lipids in meat (67).

## 6. Effects on gene transcription

Dietary nutrients may modify gene and protein expression and metabolism directly or indirectly (109, 110). Diet is one of the external factors that can influence directly the expression of genes through the biologically-active nutrients contained, which interact with transcriptional factors to positively or negatively affect signal transduction pathways (111–113). Moreover, bioactive molecules in food and feed can have an impact on epigenetics, e.g., methylation of DNA and modifications occurring in histones (111). Transcription factors are found in organs which are metabolically active, like liver, adipose tissue and intestines. Their function consists in acting as molecular sensors through a modification of gene transcriptions as a response to changes in nutrient composition (109). Polyphenols can affect various transcription factors and gene expression (114).

Due to their antioxidant properties, polyphenols can inhibit the negative consequences of excessive ROS production. Two essential regulatory mechanisms inside the cells are the gene expression regulation and the adaptive homeostasis, both of which are redox-induced (115). When the oxidation rate is high, a stress response started inside the cell to control free radicals excess and support redox homeostasis (115). Specifically, stress situations stimulate the translocation of transcription cellular factors [like nuclear factor kappa-light-chain-enhancer of activated B cells (NF-κB)] inside the nucleus; they then bind to specific DNA sites to exert a protective function, but can often exert the opposite effect (115). Oxidative stress induces different cellular processes linked to inflammation, proliferation and apoptosis (116). Polyphenols act by reducing ROS and, consequently, inhibit NF-κB, the most important regulator of the transcription of inflammatory markers (interleukins, tumor-necrosis factor etc). Cappelli et al. (116) showed for the first time the downregulation of the tumor necrosis factor-alpha (*TNFA*), advanced glycosylation end-product specific receptor (*AGER*) and BCL2-associated X apoptosis regulator (*BAX*) genes in rabbit liver after dietary supplementation with OMWW polyphenols. These effects suggest a possible inhibition from polyphenols of the effect of oxidative stress on NF-κB. Dietary OMWW also decreased *BAX* expression in rabbit’s ovary system, which highlighted the beneficial action of polyphenols on reproduction linked to inhibition of apoptosis (117).

Additionally, Sabino et al. (93) demonstrated that the incorporation of OMWW into broiler chicken diets modulated, in their jejunum, the expression of innate immune response genes against viral infections [recombinant inhibitory subunit of NF kappa B Epsilon (*IKBE*), Toll-like receptor 3 (*TLR3*), eukaryotic translation initiation factor 2 alpha kinase 2 (*EIF2AK2*), oligoadenylate synthetase like (*OASL*), myxovirus resistance gene (*MX*) and radical S-adenosyl methionine domain containing 2 (*RSAD2*)]. The same study supported that an OMWW-supplemented diet could regulate sterol biosynthesis and lipid metabolism by downregulating farnesyl diphosphate synthase (*FDPS*), matrix metalloproteinase 1 (*MMP1*) and fatty acid binding protein 3 (*FABP3*) expression in chicken small intestine (93). This nutritional strategy reduced fatty acid transportation as well as body fat accumulation in chickens (93). A few genes involved in lipid metabolism [acetyl CoA carboxylase (*ACC*) and fatty acid synthase (*FAS*)] had an upregulated expression in the serum of laying hens fed with a diet supplemented with olive cake (118). Furthermore, olive oil increased the expression of many genes encoding for heat shock proteins in broiler chickens, improving their tolerance to heat stress (119).

## 7. Conclusion

The supplementation of co-products from olive oil extraction–olive pomace, olive cake, OMWW, olive pulp and olive leaf–in monogastric animal nutrition is advisable, as these co-products are found to be harmless, sustainable and are sources of several valuable bioactive compounds. In particular, olive co-products retain the majority of EVOO polyphenols, which are the secondary metabolites most studied and recovered, given their multifunctional effects (antioxidant, antimicrobial, and anti-inflammatory) widely demonstrated in both humans and animals. Additionally, the use of these co-products in animal diets represents an innovative and efficient strategy which contributes to the circular economy, ensuring economic and environmental improvements. The suitability of co-products depends on their specific chemical features. Co-products such as OMWW can be added more easily in monogastric diets compared to others (such as olive pomace, olive pulp and olive leaf), which contain a high level of structural carbohydrates and decrease digestibility and palatability in poultry. Furthermore, the beneficial functions of the phenolic compounds contained in olive co-products and the derived extracts are related to many factors including type, dosage, absorption and metabolism. This review suggests that dietary supplementation with olive oil co-products may improve animal health, productive performances and meat quality characteristics, reduce the adverse effect of lipid peroxidation and improve the antioxidant status.

## Author contributions

FF: Writing – original draft. JT: Writing – original draft. KC: Writing – review & editing. MT-M: Writing – review & editing.
